# MIWE: detecting the critical states of complex biological systems by the mutual information weighted entropy

**DOI:** 10.1186/s12859-024-05667-z

**Published:** 2024-01-27

**Authors:** Yuke Xie, Xueqing Peng, Peiluan Li

**Affiliations:** 1 School of Mathematics and Statistics, Henan University of Science and Technology, Luoyang, 471000 China; 2 Key Laboratory of Systems Health Science of Zhejiang Province, School of Life Science, Hangzhou Institute for Advanced Study, University of Chinese Academy of Sciences, Hangzhou, 310024 China

**Keywords:** Dynamic network biomarker (DNB), Mutual information, Critical state, Differential entropy

## Abstract

Complex biological systems often undergo sudden qualitative changes during their dynamic evolution. These critical transitions are typically characterized by a catastrophic progression of the system. Identifying the critical point is critical to uncovering the underlying mechanisms of complex biological systems. However, the system may exhibit minimal changes in its state until the critical point is reached, and in the face of high throughput and strong noise data, traditional biomarkers may not be effective in distinguishing the critical state. In this study, we propose a novel approach, mutual information weighted entropy (MIWE), which uses mutual information between genes to build networks and identifies critical states by quantifying molecular dynamic differences at each stage through weighted differential entropy. The method is applied to one numerical simulation dataset and four real datasets, including bulk and single-cell expression datasets. The critical states of the system can be recognized and the robustness of MIWE method is verified by numerical simulation under the influence of different noises. Moreover, we identify two key transcription factors (TFs), CREB1 and CREB3, that regulate downstream signaling genes to coordinate cell fate commitment. The dark genes in the single-cell expression datasets are mined to reveal the potential pathway regulation mechanism.

**Supplementary Information:**

The online version contains supplementary material available at 10.1186/s12859-024-05667-z.

## Background

The development of complex disease systems can be categorized into three stages [1]: normal state, critical state and disease state. The human system has high elasticity and strong robustness in normal state and disease state. In the critical state, the human system is unstable and reversible, with low rebound and weak robustness. If the system is disturbed at this time, it may transition to the subsequent stable state or revert to the preceding stable state. Most diseases are discovered at this stage of the onset of symptoms. Despite receiving appropriate treatment, returning to a normal state remains challenging [2]. Being able to identify critical states of complex diseases at an early stage and identify tipping points before serious complications occur allows for more precise personalized treatment. In experiments conducted at the single-cell level, cell fate commitment marks a pivotal transition, and the essential endeavor of understanding and foreseeing this shift is crucial for tailoring disease models and performing personalized assessments of therapeutic efficacy in individual patients [3]. Therefore, it holds significant biomedical importance to describe the dynamic features of biological systems and accurately detect the critical stages.

In the study of complex biological systems, researchers had made great achievements in the detection of preliminary alerts of complex systems by using dynamic network markers, differential network and network entropy. The recently proposed DNB concept theoretically derived a DNB-based indicator that acts as a basis for detecting the approach of critical state [2]. Single-cell graph entropy quantified the robustness and pivotal nature within gene regulatory networks between cellular communities and could be used to provide key signals of cell fate determination [4]. At the small-sample level, evaluating the critical state can also be achieved by calculating the network entropy difference generated by perturbation using a single perturbed sample [5].

Although many studies had contributed to the development of areas related to warning signs of qualitative changes in detection systems, a large amount of research was currently conducted on bulk datasets. Compared with traditional bulk omics information, single-cell analysis is impacted by high dimensionality, noise, sparsity, and heterogeneity in samples. Characterizing the dynamics of biological systems from single-cell datasets and accurately detecting critical state is a complex task.

In this research, we suggest a differential entropy method utilizing mutual information network, i.e., mutual information weighted entropy (MIWE), which uses the differential entropy information of each stage to detect the critical state. The gene expression is transformed into probability distribution and the mutual information network is constructed at each stage. Then, according to the weight between genes in each stage network, the weighted differential entropy of each local network is calculated to quantitatively describe the fluctuations of the system at each stage, thus identifying the critical state. The MIWE method is utilized on a numerical simulation dataset and four real biological datasets, encompassing bulk sequencing and single-cell RNA sequencing (scRNA-seq) data. We effectively identify critical states of colon adenocarcinoma (COAD) and thyroid carcinoma (THCA). In addition, signals related to cell fate commitment are detected in datasets related to cell differentiation, encompassing mouse embryonic fibroblast (MEF) to neuron and mouse embryonic stem cell (mESC) to mesoderm progenitor (MP). The predicted results align with the original experimental results, which support the validity and stability of the MIWE method.

The MIWE method offers a reliable way for identifying critical states in the evolution of the complex biological systems. This approach possesses the following four benefits: (1) From the perspective of continuous variables, MIWE method can more accurately describe the mutual influence between genes than discrete variables, and can capture small changes and trends when dealing with complex data structures and nonlinear relationships, with strong robustness. (2) MIWE method is suitable for both bulk and single-cell expression data. By using edge weights to calculate phase entropy and make full use of network information, MIWE method can accurately reflect the dynamics and complexity of system changes and enhance effectiveness. (3) Based on MIWE method, critical states can be detected before critical qualitative changes occur in complex biological systems and the signaling genes of the critical state can be detected. (4) Based on the MIWE method, key TFs related to embryonic differentiation and more potential dark genes that are not detectable by traditional biomarkers are discovered. Although these dark genes are non-differential signaling genes, they have been demonstrated to participate in embryonic differentiation processes through functional pathway mechanisms.

## Methods

### Data progression and functional analysis

The MIWE method has been utilized on a numerical simulation dataset and four real biological datasets, encompassing bulk sequencing data including COAD and THCA from The Cancer Genome Atlas (TCGA) database (http://cancergenome.nih.gov) and scRNA-seq data (embryonic differentiation of MEF to neurons (GEO: GSE67310) [6] and mESC to MP (GEO: GSE79578) [7]. from the NCBI GEO database (http://www.ncbi.nlm.nih.gov/geo).

The functional annotation analysis relies on the DAVID Bioinformatics Resources (https://david.ncifcrf.gov/) and Circos (http://www.circos.ca/). Potential upstream regulators of signaling genes are identified based on ChEA3 (https://amp.pharm.mssm.edu/chea3/). Protein–Protein Interaction (PPI) networks are constructed utilizing STRING (https://string-db.org/) and the client software Cytoscape (https://cytoscape.org/).

### Theoretical background

The dynamic change of complex biological system can be regarded as irregular process, which will undergo qualitative change when approaching the critical stage. DNB theory proposed that when system approaches critical point, a set of genes or protein molecules, known as the DNB group, emerges that fulfills the following conditions: the connection between any two molecules in the DNB group swiftly grows, while the correlation with any other non-DNB molecule declines. The standard deviation of any member of the DNB group grows sharply. The system state may show small significant changes before reaching the critical point, and traditional biomarkers or methods cannot successfully predict the critical state, while the DNB index acts as a basis for identifying the approach of critical state [2]. Therefore, it is the active changes in molecular binding and spatial fluctuations, instead of differences in gene expression, that lead to differences in biological systems [8].

MIWE method transforms gene expression into probability distribution and constructs mutual information network at each stage. The edge between genes in each local network is used as the weight to calculate the weighted differential entropy of each stage. The dynamic difference changes of each stage can be measured by the difference of entropy value. The global MIWE score at every stage functions as a precursor signal for identifying the critical state.

### Algorithm to detect the tipping point based on MIWE

**Fig.1:**
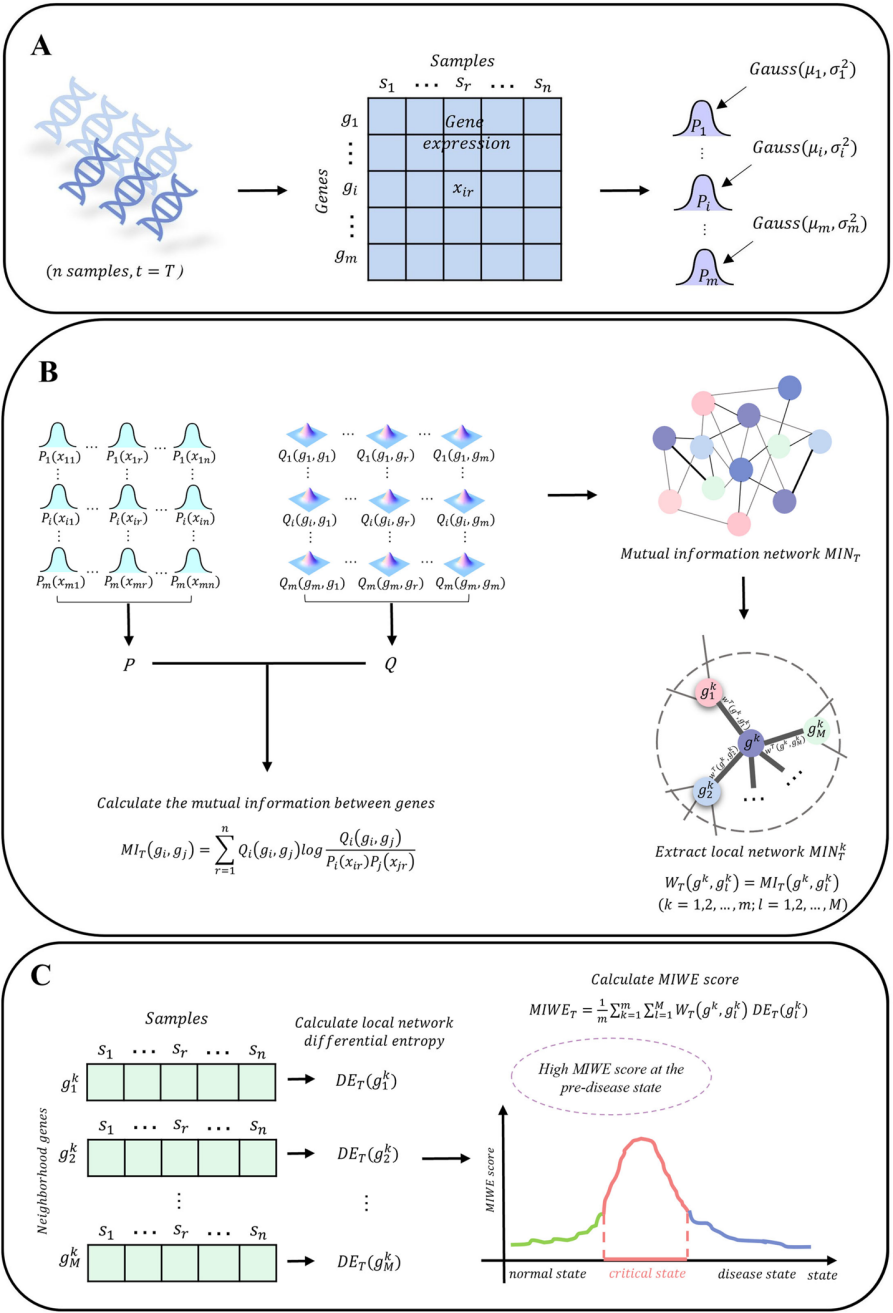
The schematic of the MIWE method. **A** Gaussian distribution is fitted for each gene. **B** Mutual information network is constructed by taking mutual information between genes as edge weight, and local network is extracted from global network. **C** The weighted differential entropy of the global network is calculated. When the system is in the critical state, the MIWE score is at a low level, and once it reaches the critical state, the MIWE score increases sharply

Given the chronological datasets of scRNA-seq or bulk sequencing, we design the following algorithm to detect the critical state (Fig. 1).

[step 1] Fit Gaussian distribution of each gene at different time $T$.

Based on given samples and transform gene expressions into probability distribution.

The Gaussian distribution is fitted according to the expression of ${g}_{i}$ $(i=\mathrm{1,2},\dots ,m)$ in the $n$ samples $\left\{{S}_{1},{S}_{2},\dots ,{S}_{n}\right\}$ at time $T$. The goodness of fit test is performed on the fitted Gaussian distribution. The gene expression values among the samples are converted to cumulative probability ${P}_{i}({x}_{ir})$. If any linear combination of genes ${g}_{i}$ and ${g}_{j}$ obey one-dimensional normal distribution, then the joint distribution between the two genes as the bivariate normal distribution, and their joint probability is ${Q}_{i}({g}_{i},{g}_{j})$.

1 $${P}_{i}\left({x}_{ir}\right)=\frac{1}{{\sigma }_{i}\sqrt{2\pi }}{\int }_{0}^{{x}_{ir}}{e}^{-\frac{{\left(u-{\mu }_{i}\right)}^{2}}{2{\sigma }_{i}^{2}}}du,$$

2 $${Q}_{i}({g}_{i},{g}_{j})=\frac{1}{2\pi {\sigma }_{i}{\sigma }_{j}\sqrt{1-{\rho }^{2}}}{\int }_{0}^{\overline{{g }_{i}}}{\int }_{0}^{\overline{{g }_{j}}}{e}^{-\frac{1}{2\left(1-{\rho }^{2}\right)}\left[\frac{{\left(u-{\mu }_{i}\right)}^{2}}{{\sigma }_{i}^{2}}-2\rho \frac{\left(u-{\mu }_{i}\right)\left(v-{\mu }_{j}\right)}{{\sigma }_{i}{\sigma }_{j}}+\frac{{\left(v-{\mu }_{j}\right)}^{2}}{{\sigma }_{j}^{2}}\right]}dudv,$$

where ${x}_{ir}$ is the gene expression values of gene ${g}_{i}$ $(i=\mathrm{1,2},\dots ,m)$ in the samples $r$ $(r=\mathrm{1,2},\dots ,n)$, $\overline{{g}_{i}}$ and $\overline{{g}_{j}}$ are the average expression values of genes ${g}_{i}$ and ${g}_{j}$ in $n$ samples at time $T$ respectively, $\rho$ is the correlation coefficient between gene ${g}_{i}$ and ${g}_{j}$ at time $T$, ${\mu }_{i}$, ${\sigma }_{i}$ $(i=\mathrm{1,2},\dots ,m)$ are the mean expression value and standard deviation of gene ${g}_{i}$ in $n$ samples at time $T$.

[step2] Construct mutual information network $MI{N}_{T}$ at each time $T$.

The edge association in the $MI{N}_{T}$ can quantitatively characterize the correlation degree between genes, in which the edge weight between genes ${g}_{i}$ and ${g}_{j}$ is determined by the $M{I}_{T}({g}_{i},{g}_{j})$ index.

3 $$M{I}_{T}\left({g}_{i},{g}_{j}\right)=\sum_{r=1}^{n}{Q}_{i}\left({g}_{i},{g}_{j}\right){\text{log}}\frac{{Q}_{i}\left({g}_{i},{g}_{j}\right)}{{P}_{i}\left({x}_{ir}\right){P}_{j}\left({x}_{jr}\right)},$$

the degree of correlation between genes is described from the perspective of information. In the presence of a certain level of gene correlation, increased mutual information is observed when there is less randomness between genes.

[step3] Extract the local network from the global network.

Extract the local network ${MIN}_{T}^{k}$ $\left(k=1,2,\dots ,m\right)$ from the global network $MI{N}_{T}$ at each time $T$, which contains a central gene ${g}^{k}$ and first-order neighbors $\{{g}_{1}^{k},{g}_{2}^{k},...,{g}_{M}^{k}\}$, where the edge weight ${W}_{T}({g}^{k},{g}_{l}^{k})=M{I}_{T}({g}^{k},{g}_{l}^{k})$ in the local network.

[step4] Calculate differential entropy of the neighborhood gene ${g}_{l}^{k}$ $(l=\mathrm{1,2},\dots ,M)$ in local network ${MIN}_{T}^{k}$ $\left(k=\mathrm{1,2},\dots ,m\right)$.

For each local network ${MIN}_{T}^{k}$ $\left(k=\mathrm{1,2},\dots ,m\right)$ at time $T$, the differential entropy of neighborhood gene ${g}_{l}^{k}$ $(l=\mathrm{1,2},\dots ,M)$ is denoted as:

4 $$D{E}_{T}\left({g}_{l}^{k}\right)=-{\int }_{0}^{\overline{{g }_{l}^{k}}}f\left(x\right){\text{log}}f\left(x\right)dx,$$

5 $$f\left(x\right)=\frac{1}{{\sigma }_{l}^{k}\sqrt{2\pi }}{e}^{-\frac{{\left(x-{\mu }_{l}^{k}\right)}^{2}}{2{\left({\sigma }_{l}^{k}\right)}^{2}}},$$

where ${g}_{l}^{k}$ are the average expression values of genes ${g}_{l}^{k}$ in $n$ samples at time $T$, ${\mu }_{l}^{k}$, ${\sigma }_{l}^{k}$ $(l=\mathrm{1,2},\dots ,M)$ are the mean expression value and standard deviation of gene ${g}_{l}^{k}$ in $n$ samples at time $T$.

[step5] Calculate mutual information weighted entropy of the global network $MIW{E}_{T}$.

Calculate the weighted entropy value $MIW{E}_{T}^{k}$ $(k=\mathrm{1,2},\dots ,m)$ of each local network at time $T$, namely,

6 $$MIW{E}_{T}^{k}=\sum_{l=1}^{M}{W}_{T}({g}^{k},{g}_{l}^{k})D{E}_{T}\left({g}_{l}^{k}\right),$$

then the weighted entropy score of the global network is:

7 $$MIW{E}_{T}=\frac{1}{m}\sum_{k=1}^{m}MIW{E}_{T}^{k},$$

Signaling biomolecules exhibit significant collective behavior and intense fluctuations during the critical transition of a complex dynamic system. The weighted entropy of the local network containing signal biomolecules in the critical state is significantly different from that in the pretransition state. If $MIW{E}_{T}$ sharply increases, then time point $T$ is the critical point, and the top 5% genes of $MIW{E}_{T}^{k}$ are signaling genes that regarded as DNBs in this work.

## Results

### Validation based on numerical simulation

We use a theoretical model to validate the robustness of MIWE method, and construct a 10-node monitoring network based on the Michaelis–Menten equation [9], which is mainly used to study transcription and translation processes [10], nonlinear biological processes [11, 12]. The 10-node monitoring network can generate datasets for numerical simulation, and as the parameter p varies from − 0.5 to 0.25, the system experiences the critical transition when the parameter value is p = 0.

**Fig.2:**
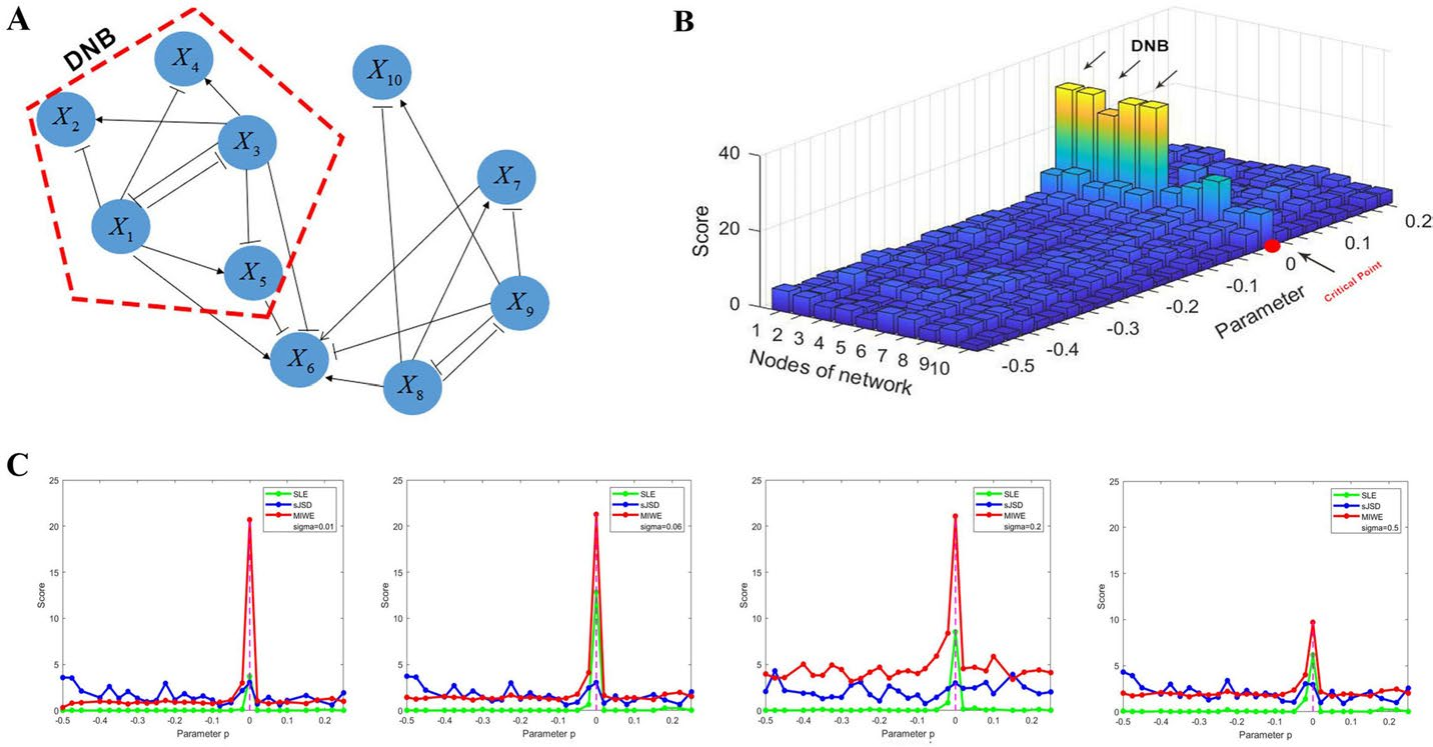
Numerical simulation dataset is used to verify the effectiveness of MIWE. **A** Gene regulatory network model, where the arrow represents positive regulation. **B** MIWE score for each parameter of 10 nodes. **C** Comparison of the robustness of the MIWE method with the SLE, sJSD method at various levels of noise strength

Figure 2A shows the gene regulatory network composed of 10 nodes with both activating and inhibitory interactions. Before the system reaches the critical point, MIWE score is at a low level. When the parameter value p = 0, MIWE score increases sharply, providing a precursor signal for the upcoming state change (Fig. 2B). Considering the existence of strong noise in real datasets, we verify the MIWE method under the influence of different noises, and compare it with SLE [5] and sJSD [13] methods (Fig. 2C). As the noise intensity increased, MIWE consistently offers early warning signals for impending tipping points with heightened sensitivity, indicating that the MIWE method is more robust and efficient in detecting critical points in biological processes. Additional information regarding the numerical simulation is available in the Additional file 1: Section A.

### Identifying cell fate commitment during embryonic differentiation

To verify the validity of the MIWE method and detect the transformation of cell fate commitment, the method is utilized on two datasets of cell differentiation, including MEF to neurons (GSE67310) and mESC to MP (GSE79578) data. The weighted entropy of each local network is calculated according to the steps of the algorithm. Finally, the average weighted entropy (Eq. 7) is taken at each time point to quantitatively characterize the criticality of the single-cell community.

**Fig.3:**
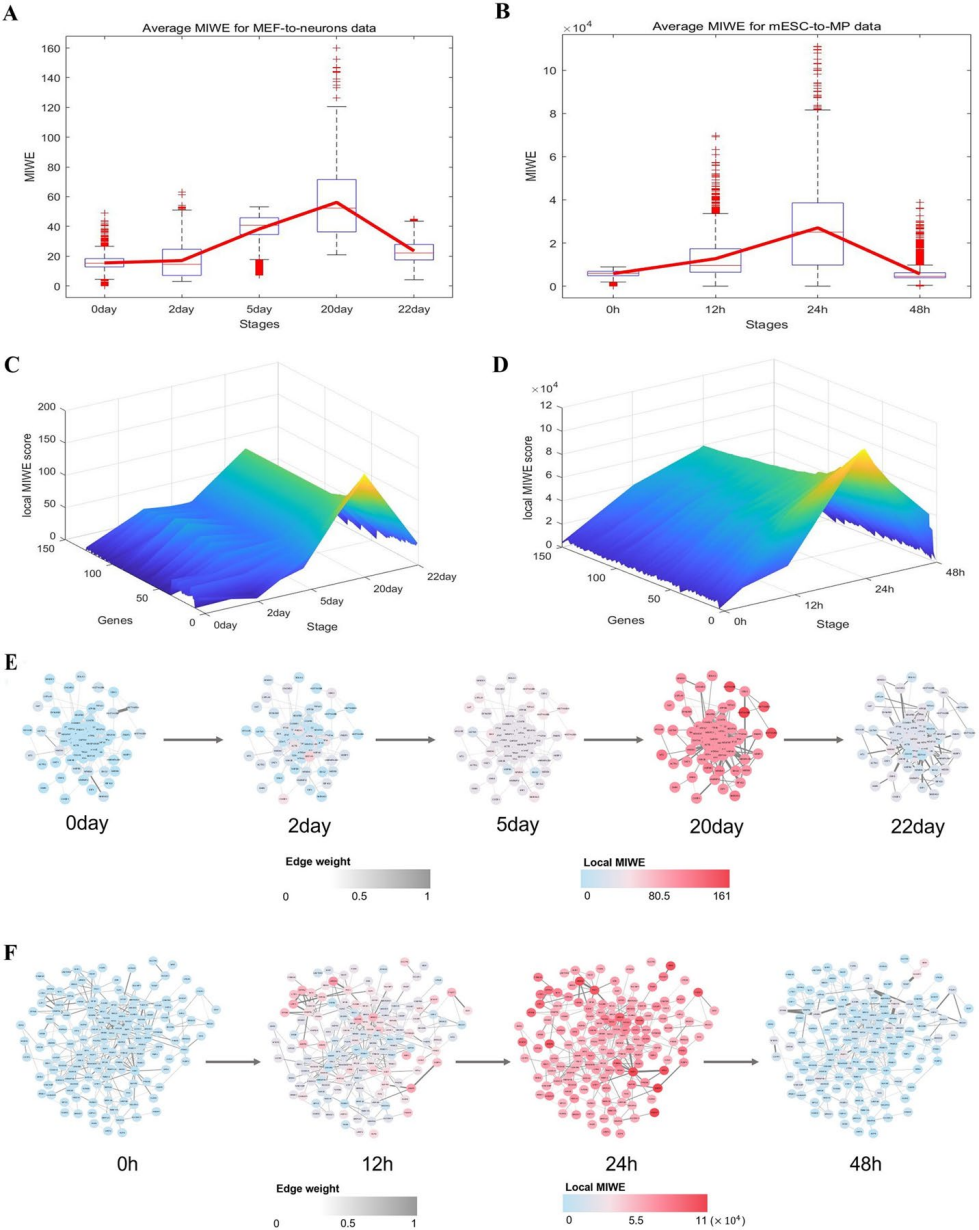
Detecting the signal of cell fate commitment. The MIWE value is calculated for **A** MEF to neurons and **B** mESC to MP. The landscape of local MIWE values illustrates the dynamic evolution of network entropy in a global view for **C** MEF-to-neuron, **D** mESC to MP. The dynamical evolution of gene regulatory networks for the **E** MEF-to-neuron, **F** mESC to MP

We use the MIWE score curve across time points to show the fluctuations of cell differentiation at each stage. For MEF to neurons data, MIWE scores increase significantly from day 5 to day 20 (Fig. 3A), providing a precursor signal for the imminent differentiation into neurons, indicating that cell fate commitment began on day 22. In mESC to MP data, MIWE scores at 24 h are significantly different from those at adjacent stages (Fig. 3B), indicating that transition is about to take place after 24 h, namely mouse embryonic stem cells differentiate into mesoderm. The algorithm detection results of the two datasets are consistent with the original experimental observation. Moreover, to prove the robustness of the proposed method, box graphs of weighted entropy at each stage are presented based on samples at each time point. The median value of the block diagram provides obvious signal for the critical point, indicating that the MIWE value is highly robust to the sample noise.

The signaling genes are identified as the top 5% of genes with the highest local MIWE scores, which may be highly correlated with cell differentiation. The landscape map shows dynamic changes in the distribution of local MIWE values of signaling genes in the global view (Fig. 3C, D), and the local MIWE values of the signaling genes in the two datasets increase sharply at day 20 and 24 h, respectively. Changes in local MIWE values of all genes are shown in Additional file 1: Fig. S2. In addition, signaling genes are mapped to PPI networks to observe the dynamic changes of networks at different stages. For both datasets, significant changes in network structure are observed at day 20 and 24 h, respectively, indicating an upcoming cell fate commitment (Fig. 3E, F).

### Detecting potential upstream TFs

**Fig.4:**
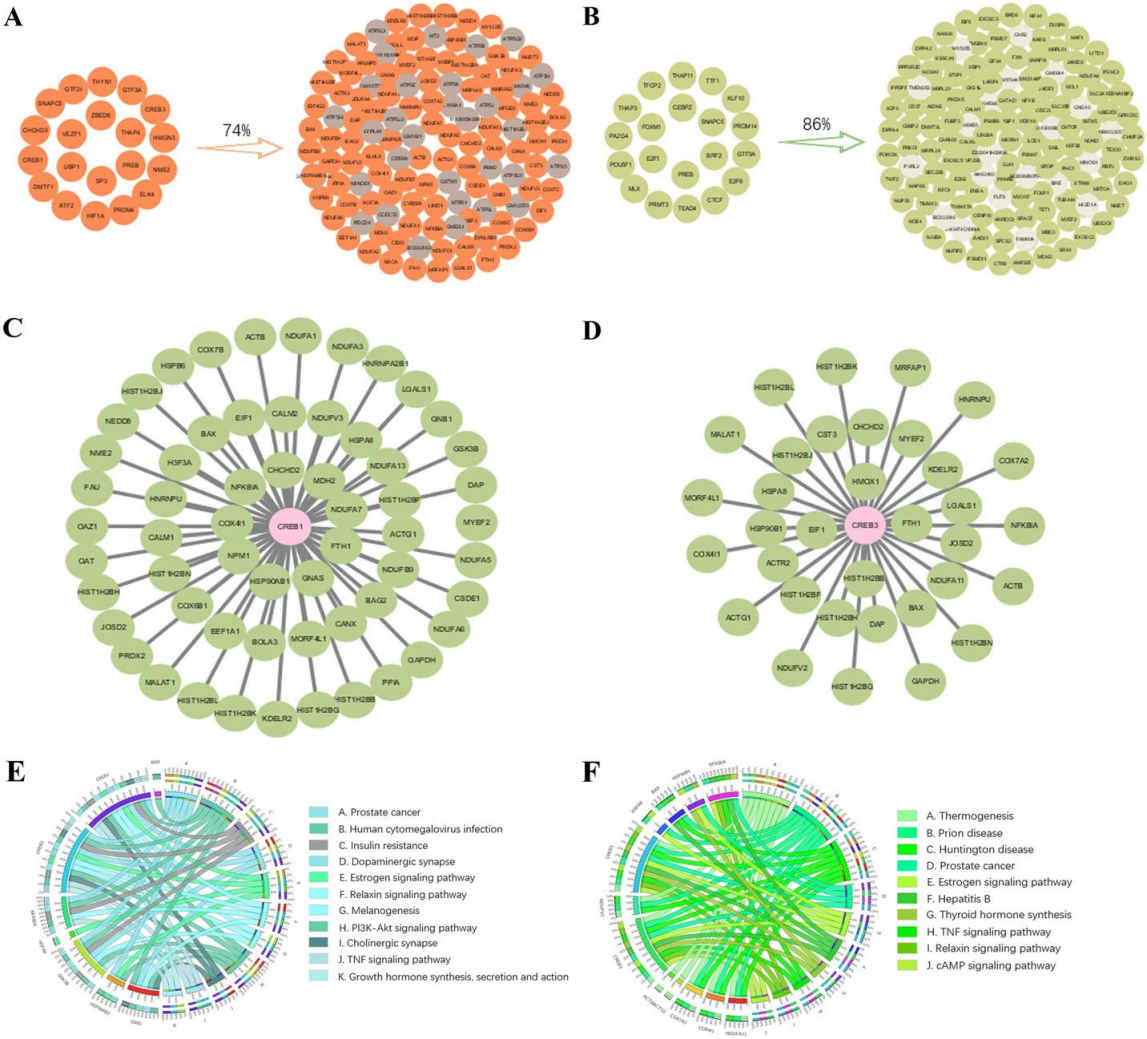
TFs regulation and related enrichment analysis. **A** TFs modulated 74% of signaling genes identified by GSE67310 critical point. **B** TFs modulated 86% of signaling genes identified by GSE79578 critical point. Regulatory network of **C** CREB1, **D** CREB3. **E** CREB1 and its regulated signaling genes participate in significant biological processes and KEGG pathways. The outer ring's left side signifies the signaling genes identified by MIWE, while the right side represents the diverse biological processes associated with these genes. The inner ring depicts various enrichment pathways, with connection color and width indicating different levels of gene function significance. **F** CREB3 and its regulated signaling genes participate in significant biological processes and KEGG pathways

TFs are important molecules that control gene expression and can be considered as key players in controlling or driving cell fate commitment [14, 15]. In order to explore the involvement of the signaling genes identified in the two cell differentiation datasets in the process of cell fate commitment, we separately predict the TFs of the two groups of signaling genes on the ChEA3 website, and select the top 20 in the comprehensive average ranking as the main research content. In the GSE67310 and GSE79578 data, two sets of TFs modulate 74% and 86% of the signaling genes at the critical point, respectively (Fig. 4A, B).

Some TFs play an important role in cell differentiation and proliferation. They are closely related to cell proliferation and self-renewal, and are crucial contributors to the early embryonic development and cell lineage specification. For GSE67310 data, the absence of CHCHD3 expression can lead to tissue undergrowth and cell proliferation defects [16], VEZF1 can regulate cell differentiation and proliferation and participate in the early vascular differentiation process [17], SP3 is required for perinatal survival in mice [18]. GTF2I indirectly contributes to the transcriptional regulation of genes controlling cell proliferation and cell cycle through encoding transcription factor TFII-I [19]. Functional annotations of TFs for GSE79578 data are in the Additional file 1: Section C.

**Fig.5:**
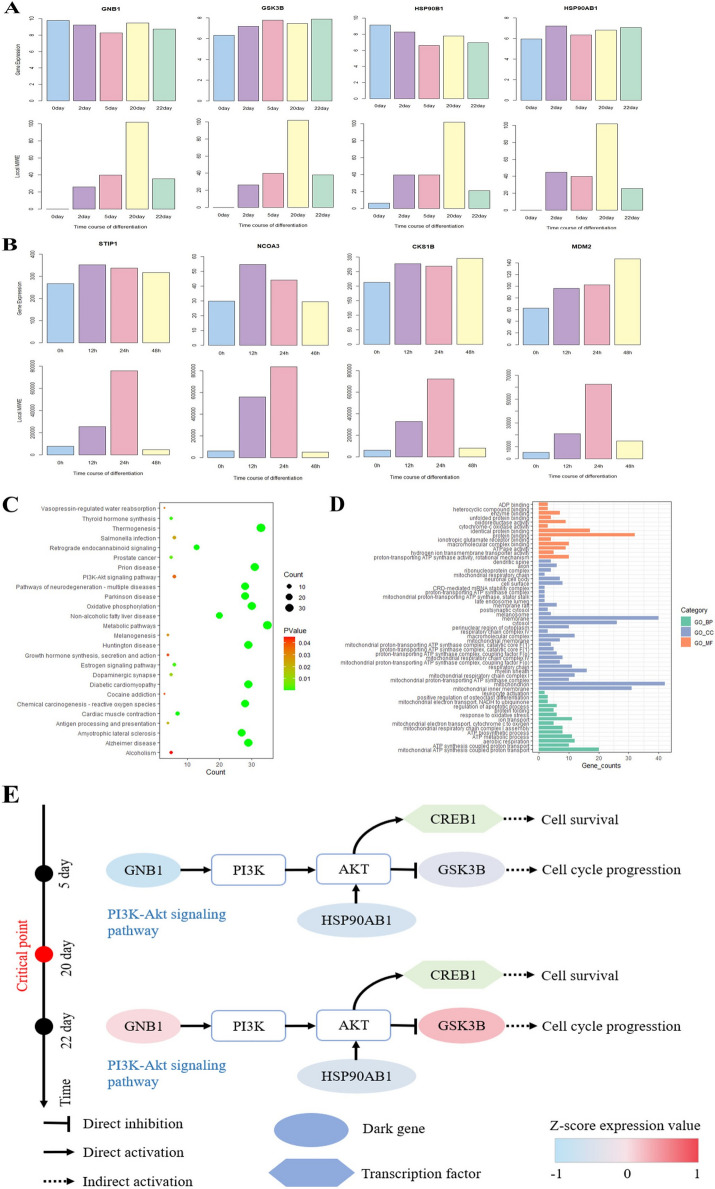
Potential regulatory mechanisms related to embryonic differentiation revealed by dark genes. Dynamic changes of gene expression and entropy of dark genes for **A** MEF to neurons, **B** mESC to MP. **C** Pathways enriched of MEF to neurons. **D** GO analysis of MEF to neurons. **E** The enrichment and regulation of related dark genes of MEF to neurons

In the analysis of TFs from GSE67310 data, we find two relatively key TFs, which can contribute to a more profound comprehension of the molecular mechanisms of embryonic development and hold significant implications for the treatment and prevention of related diseases, namely CREB1 and CREB3. CREB1 plays a role in cell proliferation, myogenic differentiation and other related pathways [20]. CREB3 is involved in embryonic development and the differentiation of other tissues and organs, such as osteoblast differentiation [21]. In order to visualize the downstream signaling genes regulated by these two TFs, we present the regulatory network centered on TFs (Fig. 4C, D). Combined with the TFs and their regulated signaling genes, we find that they are involved in some signaling pathways related to embryonic differentiation (Fig. 4E, F). The TNF signaling pathway is central to a range of physiological and pathological processes, influencing cell proliferation, differentiation, apoptosis, immune response regulation, and inflammation induction. Activation of TNF signaling pathway can trigger activation of PI3K-Akt signaling pathway. The interaction between CREB1 and NF-κB can modulate the transcription of downstream genes and thus contribute to the control of apoptosis and other processes. The mechanism of CREB1 in the PI3K-Akt signaling pathway is shown in Fig. 5E. The cAMP signaling pathway governs various intracellular processes, such as the modulation of cell proliferation, differentiation, and apoptosis via the activation of cAMP-dependent protein kinase (PKA) [22]. Phosphorylated PKA can then further phosphorylate CREB3 and activate its transcriptional activity. By binding to CBP, CREB3 regulates the transcription of specific genes and thus contributes to the control of various cellular physiological responses. In this way, CREB3 is crucial for cell growth and development, metabolic regulation, and stress response.

### The underlying signaling mechanisms revealed by dark genes based on scRNA-seq data

Differential expression not only helps to reveal the secret of biological process, but also provides important theoretical basis for gene diagnosis and therapy. In many medical experiments and molecular studies, differentially expressed genes (DEGs) serve as markers or drug therapeutic targets, while some non-differentially expressed genes (non-DEGs) are often ignored, which will also have a significant role in biological processes and may be potential therapeutic biomarkers. In this study, genes with no differential expression but sensitive to the MIWE score are defined as dark genes, and differential MIWE analysis is performed on the two embryonic differentiation datasets to show the differences in MIWE values and gene expression of dark genes in the two datasets (Fig. 5A, B). There is a clear observation that gene expression remains relatively constant at each stage, while there are significant differences in MIWE values.

For mESC to MP data, it has been confirmed that some dark genes are closely related to embryonic differentiation, which are mainly involved in the regulation of chemical reactions in cells or organisms, macromolecular metabolism, and the frequency, rate or degree of gene expression and other biological processes. Extracellular STIP1 engages with diverse receptors to boost induced differentiation, cell proliferation, and protein synthesis [23]. Low expression of Receptor coactivator 3 (NCOA3) may lead to decreased differentiation potential of embryonic stem cells in vitro and in vivo [24]. CKS1B regulates cell cycle processes by engaging with cyclin-dependent kinase (CDK) and SCF complex to affect cell proliferation [25]. MDM2, an E3 ubiquitin ligase, plays a crucial role in the differentiation of various cell types, including osteoblasts and myoblasts [26].

To investigate the potential signaling mechanisms indicated by mouse dark genes and their domain genes, we conduct a series of functional analyses of dark genes from MEF to neurons (Fig. 5C, D). HSP90B1 participates in the Thyroid hormone synthesis pathway, in which synthetic thyroid hormones bind to nuclear receptors and control the expression of numerous genes associated with cell cycle regulation and differentiation [27]. In Prostate cancer pathway, HSP90AB1 and HSP90B1 can indirectly affect cell proliferation and survival by activating Ar and thus binding to DNA sites. GSK3B phosphorylates β-catenin to further activate Cyclin D1, an important regulatory factor of cell cycle [28], it can also lead to cell proliferation. The PI3K-Akt signaling pathway serves as a crucial hub governing cell growth, proliferation and metabolism in mammalian cells [29]. Figure 5E shows the potential mechanism of dark genes in MEF to neurons data and their domain genes in pathways. During embryonic differentiation, the high expression of GNB1 activates PI3K, which is then combined with HSP90 to activate the downstream target AKT of PI3K, HSP90 regulates various biological processes, such as cell growth, differentiation, and survival [30], AKT kinase translates diverse signals into intracellular cues governing cell survival, proliferation, metabolism, and differentiation [31] and transmits them to downstream genes, affecting cell proliferation and differentiation. The gene expression of the dark genes changes significantly between day 5 and day 22, and the recognized critical point could serve as a crucial time point to guide the differentiation of MEF to neurons.

### Identifying the critical state during cancer progression

**Fig.6:**
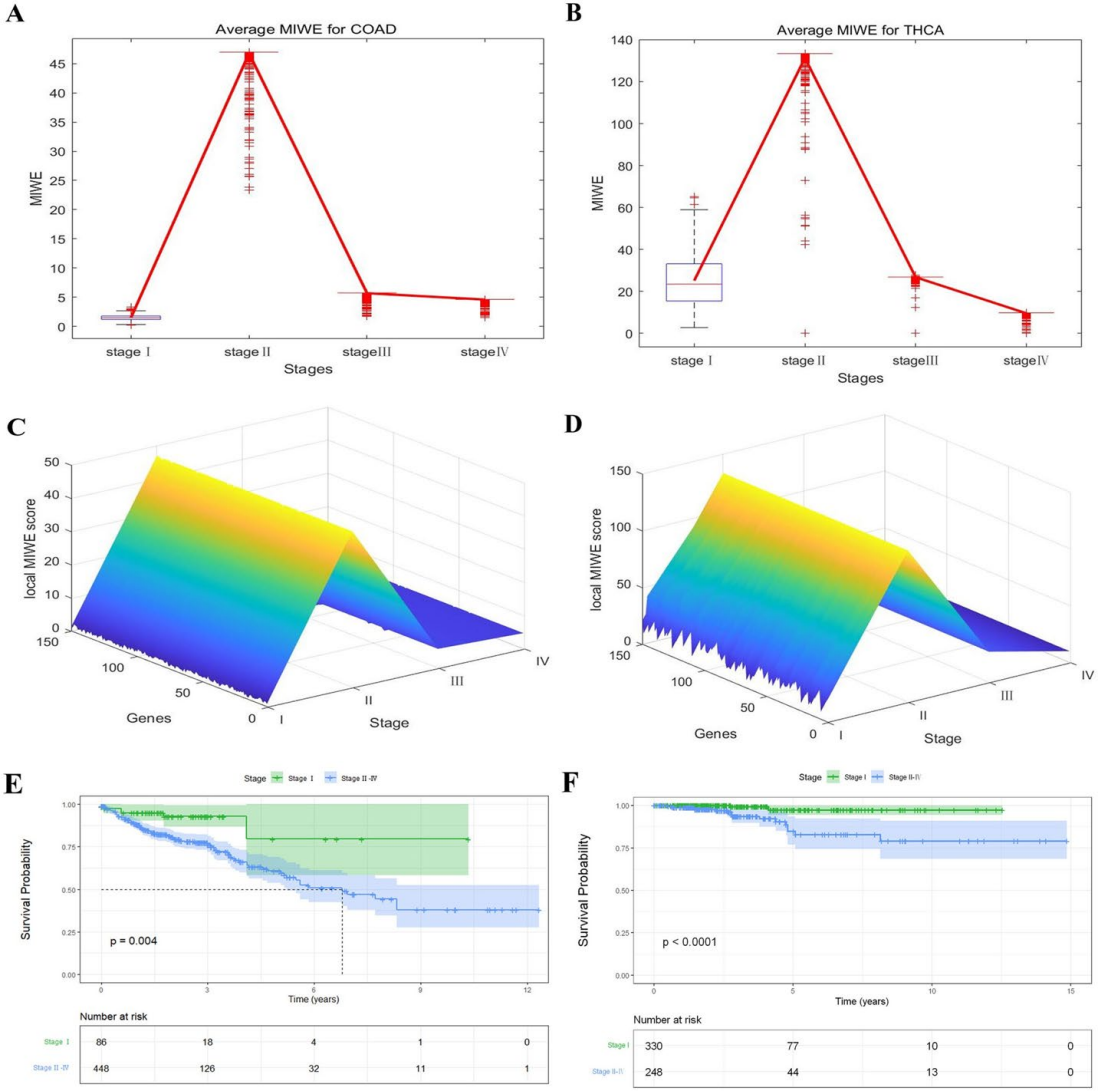
Detection of the critical point of cancer progression. The MIWE score for **A** COAD, **B** THCA. Landscapes of the local MIWE score for **C** COAD, **D** THCA. Survival analysis before and after the identified critical states for **E** COAD, **F** THCA

In addition to identifying the critical transition of embryonic differentiation, we also apply MIWE algorithm to two cancer datasets, COAD and THCA, and take healthy samples as the reference group to participate in the entropy calculation at each stage. In the second phase, local MIWE values in the COAD and THCA data increased significantly (Fig. 6A, B), which could be identified as a critical state of disease progression. The landscape map shows the dynamic changes of local MIWE values of signaling genes (Fig. 6C, D), which also indicated the abnormal system in the second stage. In addition, genes with the top 5% maximum local MIWE value at the critical stage are used as signaling genes, Changes in local MIWE values of all genes and dynamic changes of signaling genes in PPI network are shown in the Additional file 1: Fig. S3. Detection of critical points before disease progression or metastasis is conducive to timely clinical intervention for subsequent treatment. MIWE method can provide early warning signals in the course of disease development, which is helpful for disease treatment.

We use the Kaplan–Meier method for prognostic survival analysis of clinical samples from two cancers. By comparing the survival rate of each sample and its standard error, it can be observed that the prognosis of patients diagnosed before the critical state is significantly different from that of patients diagnosed after the critical stage, with P values less than 0.05 (Fig. 6E, F). Patients treated before deterioration have higher survival rate and longer survival time. More details of survival analysis are shown in the Additional file 1: Section E.

### Functional analysis of the common MIWE signaling genes among two cancers

**Fig.7:**
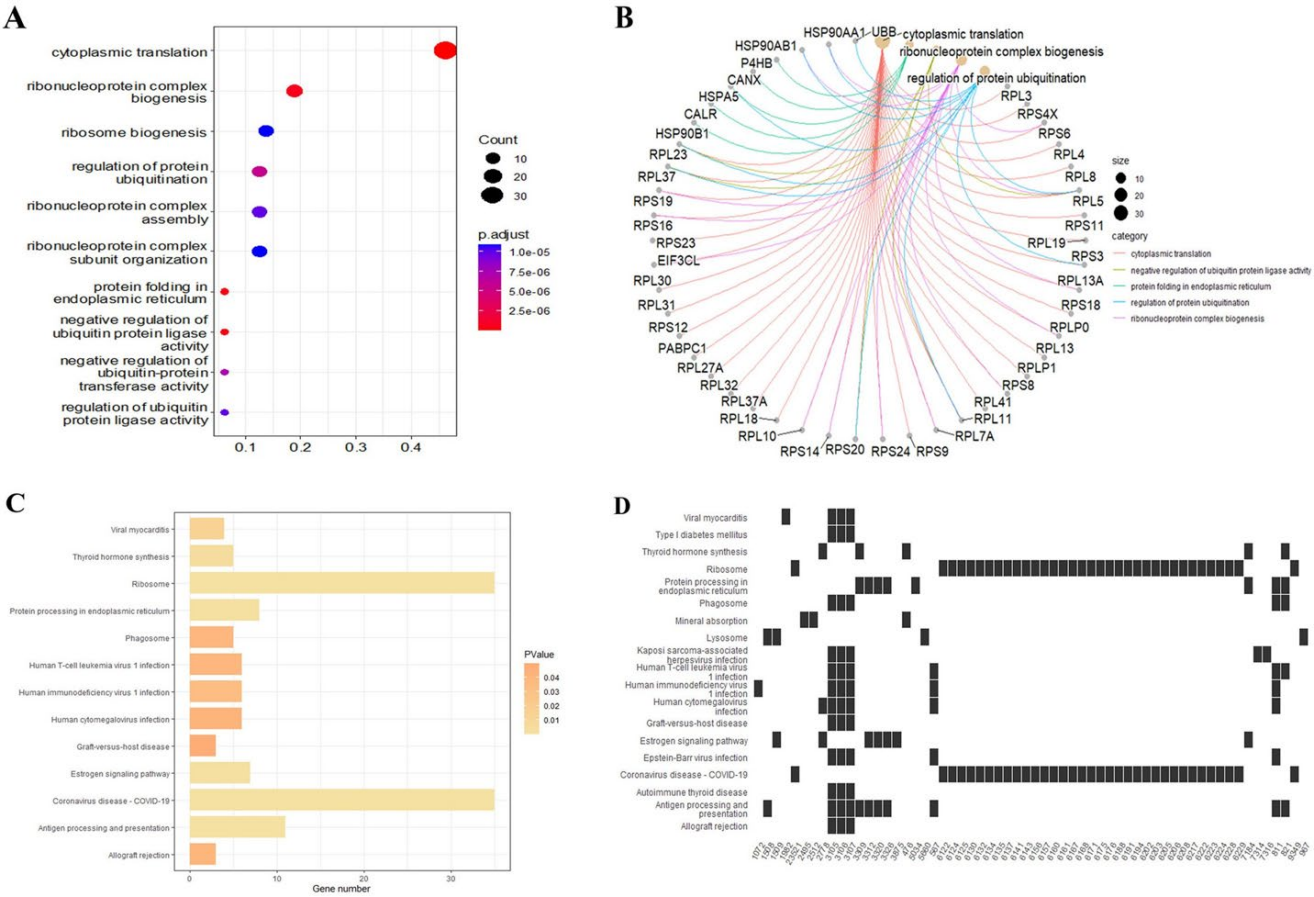
Functional analysis of common signaling genes in two cancers. **A** Common signaling genes involve in major biological processes. **B** The association of genes with biological processes. **C** Common signaling genes involve in cancer related pathways. **D** The association between genes and pathways, where the number represents the ENTREZ ID of the gene

To comprehend the mechanism of signaling genes involved in disease development, we perform functional enrichment analysis of the common signaling genes of two cancers. The GO analysis results show that the signaling genes are mainly involved in the chemical reaction of protein formation in the cytoplasm, the macromolecular modification process of synthesis or assembly of ribonucleoprotein complexes, and the regulation of the rate of ubiquitin groups added to proteins (Fig. 7A). The lack of numerous ribosomal proteins can directly impact the overall translation process and the global expression of proteins, contributing to the onset of various diseases, including cancer [32]. Figure 7B shows the association between genes and biological processes. Elevated in numerous solid tumors, HSP90AB1 is believed to stimulate angiogenesis and facilitate cancer metastasis [33]. Heat shock protein family A (HSPA5) as a diagnostic and prognostic biomarker for various malignancies [34]. P4HB can influence tumor formation in a collagen-dependent or collagen-independent manner [35].

In addition, common signaling genes are involved in several pathways associated with cancer progression (Fig. 7C). MHC Class I and Class II antigen processing and presentation pathways present peptides to circulating CD8 + cytotoxic T cells and CD4 + helper T cells, respectively, to recognize pathogens and transform cells. Immune surveillance of transformed cells/tumor cells induces alterations in antigen processing and presentation pathways to evade immune response, which is an important process in tumor development [36]. Figure 7D shows the related pathways involved in each gene. β2-microglobulin (B2M) plays a physiological and pathological role in tumor cells [37]. In Antigen processing and presentation, the complex of B2M and HLA-B/C activates down-stream signals, upregulates and enhances T cell immunity, and plays an important role in controlling colon/rectal cancer growth [38]. Studies have shown that B2M is a potential tumor suppressor gene in COAD and has been identified as a potential biomarker for THCA [39]. Processing, modification, and folding of proteins in the endoplasmic reticulum (ER) are highly regulated procedures that dictate cell function, fate, and survival. Abnormal activation of the downstream signaling pathway of ER has been proven to be a key regulatory factor for tumor growth and metastasis [40]. Estrogen can affect tumor progression by regulating tumor microenvironment and plays a pivotal role in the occurrence and development of THCA [41]. GNAS is considered to be an oncogene that can be constitutionally activated by a specific point mutation of Guanine nucleotide binding protein alpha subunit (Gsα) in the Estrogen signaling pathway, thus activating multiple cancer-related pathways [42].

## Discussion

Identifying critical states in complex biological systems is essential, such as critical stages of disease progression and cell fate commitments during embryonic development, early warning signs of disease progression that can prepare for treatment, and understanding cell fate commitment that can build individual specific disease models. However, identifying critical transitions in complex biological systems is often challenging, and real biological datasets have strong noise and cannot characterize the dynamics of biological processes. In this study, we propose MIWE method for identifying cell fate transitions and complex disease critical states. The MIWE score quantifies the dynamic differences of mutual information networks at each stage based on weighted differential entropy at each time point, and converts gene expression values into probabilities to minimize the influence of strong noise. To verify the validity of the MIWE algorithm, the method is utilized on one simulated dataset and four real datasets, encompassing two scRNA-seq datasets and two bulk sequencing datasets.

Based on the MIWE method, we successfully detect the critical states the dynamic processes of complex biological systems. The function analysis of signaling genes in critical stage reveals the important role of signaling genes in embryonic differentiation or cancer development. In addition, we focus on exploring the potential signaling mechanisms of some non-differential signaling genes in embryonic differentiation pathways. Although they are not DEGs, the pathways involve are highly related to cell differentiation.

MIWE method is model-free and suitable for both bulk and single-cell expression data. However, MIWE also has limitations, as undirected networks are used in the construction of networks, which ignore causal relationships between nodes compared with directed networks. In addition, the joint distribution of two genes is binary normal distribution if and only if any linear combination of them follows a normal distribution. In general, the MIWE method helps to identify and detect critical states in complex biological systems, providing a theoretical basis for timely clinical intervention and disease modeling.

## Conclusions

In this study, we propose a new method, mutual information weighted entropy (MIWE), which identifies critical states by quantifying the molecular dynamic differences at each stage by calculating the weighted differential entropy of each stage of the global network. The robustness of the proposed method under the influence of different noises is verified by numerical simulation. In addition, we identify two key transcription factors (TFs), CREB1 and CREB3, which are involved in cell proliferation and differentiation by regulating downstream signaling genes. The dark genes in the single-cell expression dataset are mined to reveal the potential pathway regulation mechanisms involved.

### Supplementary Information

**Additional file 1. **Supplementary materials, figures, tables.

## Data Availability

To ensure reproducible results, all data can be found here: http://www.ncbi.nlm.nih.gov/geo/ and http://cancergenome.nih.gov, and the original code are available at https://github.com/xykxingchen/MIWE.
