# Supplementary material for: MIWE: detecting the critical states of complex biological systems by the mutual information weighted entropy
Source: BMC Bioinformatics. 2024 Jan 27;25:44. doi: 10.1186/s12859-024-05667-z (PMC10822190; doi:10.1186/s12859-024-05667-z)
Supplement: Supplementary file 1 — Additional file 1. Supplementary materials, figures, tables. [file 12859_2024_5667_MOESM1_ESM.docx]

**Supplementary Material for**

**“ MIWE: Detecting the critical states of complex biological systems by the mutual information weighted entropy”**

Yuke Xie^1,2^, Xueqing Peng^1^, and Peiluan Li^1,^*

^1^ School of Mathematics and Statistics, Henan University of Science and Technology, Luoyang, 471000, China

^2^ Key Laboratory of Systems Health Science of Zhejiang Province, School of Life Science, Hangzhou Institute for Advanced Study, University of Chinese Academy of Sciences, Hangzhou 310024, China

*Corresponding authors: Peiluan Li, E-mail: 15038522015@163.com.

**Contents**

[A. The details of numerical simulation 2](#_Toc156641171)

[B. Changes in local MIWE values for each gene in four real datasets 5](#_Toc156641172)

[C. Functional annotation of TFs associated with embryonic differentiation from GSE79578 data 6](#_Toc156641173)

[D. Dynamic evolution of PPIs for two TCGA cancers: COAD, THCA 7](#_Toc156641174)

[E. The Kaplan-Meier survival analysis of two cancer datasets 8](#_Toc156641175)

[F. Leave-one-out cross validation 9](#_Toc156641176)

[References 10](#_Toc156641177)

# The details of numerical simulation

To verify the robustness of MIWE method in the numerical simulation dataset, we use a regulatory network with 10 genes (Fig. S1) to detect the critical state. These types of gene molecular regulatory networks are often used to study transcription and translation [1-3], and various nonlinear biological processes [4-6]in gene regulation.


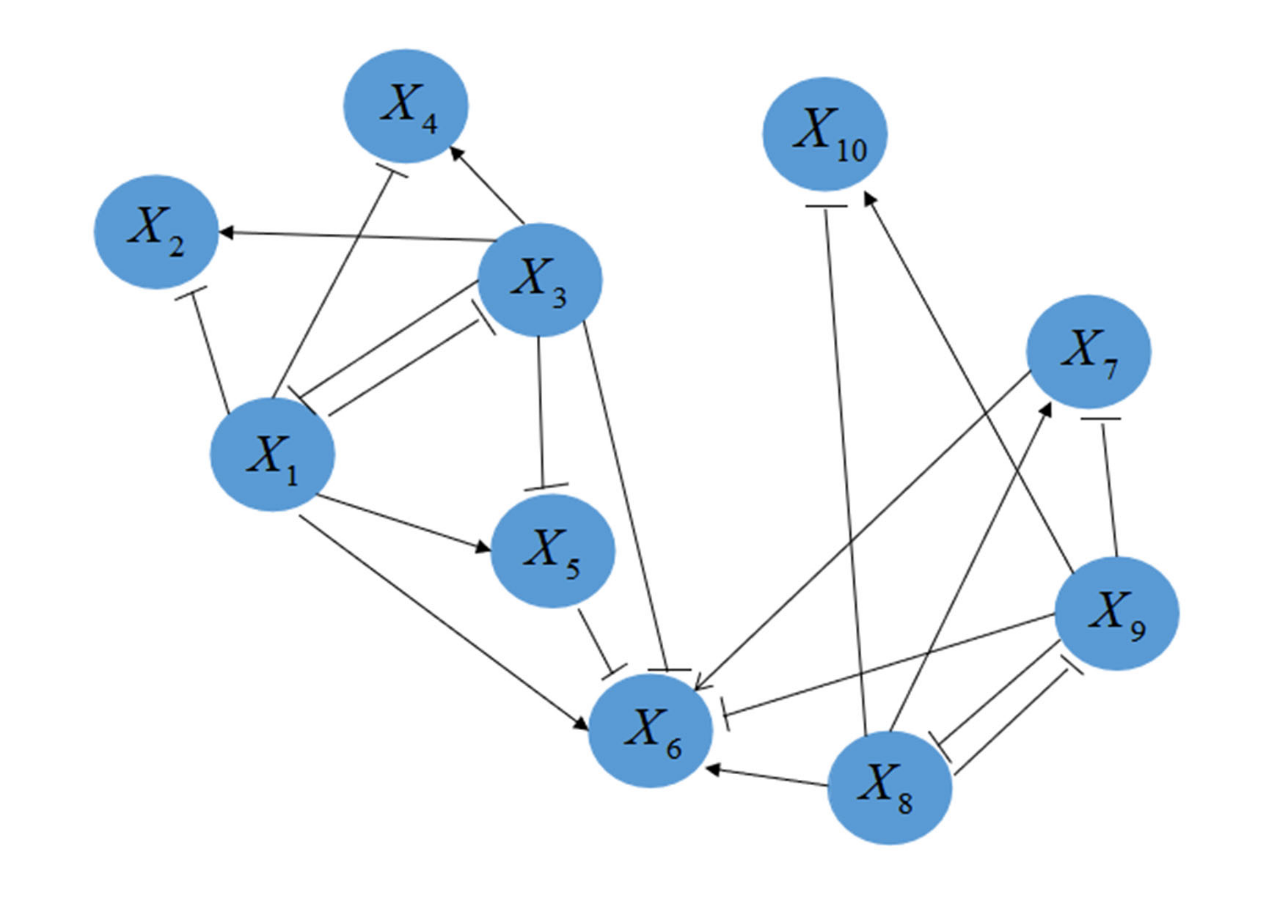


**Fig. S1.** **A model of a ten-molecular network.** The edges represent positive or negative regulations among nodes.

Ten differential equations in following Eq. (S1) represent the gene regulation of ten genes in the network. In the network, gene regulation is represented in a Michaelis-Menten form with the degradation rates, which are linearly proportional to the concentrations of the corresponding genes.

$\left\{ \begin{aligned} &\frac{\begin{aligned} &dz_{1}(t) \end{aligned}}{dt}=\frac{3\left| P \right|-8}{22}+\frac{8-3\left| P \right|}{22(1+z_{3}(t))}-\frac{8+3\left| P \right|}{22}z_{1}(t)+\xi_{1}(t) \\ &\frac{dz_{2}(t)}{dt}=\frac{3\left| P \right|-6}{22}+\frac{6-3\left| P \right|}{22(1+z_{1}(t))}+\frac{(6-3\left| P \right|)z_{3}(t)}{22(1+z_{3}(t))}-\frac{6}{11}z_{2}(t)+\xi_{2}(t) \\ &\frac{dz_{3}(t)}{dt}=\frac{3\left| P \right|-8}{22}+\frac{8-3\left| P \right|}{22(1+z_{1}(t))}-\frac{8+3\left| P \right|}{22}z_{3}(t)+\xi_{3}(t) \\ &\frac{dz_{4}(t)}{dt}=\frac{3\left| P \right|-10}{22}+\frac{10-3\left| P \right|}{22(1+z_{1}(t))}+\frac{(10-3\left| P \right|)z_{3}(t)}{22(1+z_{3}(t))}-\frac{10}{11}z_{4}(t)+\xi_{4}(t) \\ &\frac{dz_{5}(t)}{dt}=\frac{3\left| P \right|-12}{22}+\frac{(12-3\left| P \right|)z_{1}(t)}{22(1+z_{1}(t))}+\frac{12-3\left| P \right|}{22(1+z_{3}(t))}-\frac{12}{11}z_{5}(t)+\xi_{5}(t) \\ &\frac{dz_{6}(t)}{dt}=-\frac{4}{11}+\frac{z_{1}(t)}{11(1+z_{1}(t))}+\frac{1}{11(1+z_{3}(t))}+\frac{2}{11(1+z_{5}(t))}+\frac{2z_{7}(t)}{11(1+z_{7}(t))}+\frac{z_{8}(t)}{11(1+z_{8}(t))}+\frac{1}{11(1+z_{9}(t))}-\frac{14}{11}z_{6}(t)+\xi_{6}(t) \\ &\frac{dz_{7}(t)}{dt}=-\frac{1}{11}+\frac{z_{8}(t)}{11(1+z_{8}(t))}+\frac{1}{11(1+z_{9}(t))}-\frac{16}{11}z_{7}(t)+\xi_{7}(t) \\ &\frac{dz_{8}(t)}{dt}=-\frac{1}{11}+\frac{1}{11(1+z_{9}(t))}-\frac{19}{11}z_{8}(t)+\xi_{8}(t) \\ &\frac{dz_{9}(t)}{dt}=-\frac{1}{11}+\frac{1}{11(1+z_{8}(t))}-\frac{19}{11}z_{9}(t)+\xi_{9}(t) \\ &\frac{dz_{10}(t)}{dt}=-\frac{1}{11}+\frac{1}{11(1+z_{8}(t))}+\frac{3z_{9}(t)}{11(1+z_{9}(t))}-2z_{10}(t)+\xi_{10}(t) \end{aligned} \right.$ (S1)

where $p$ is a scalar control parameter and ${}_{i}(t)(i = 1, 2, ..., 10)$ are Gaussian noises with zero means and covariances $k_{ij}=Cov({}_{i},{}_{j})$. $z_{i}(t)(i = 1, 2, ..., 10)$ represent the concentrations of mRNA-$i$. In Eq.(S1), there is the degradation rates of mRNAs $\text{R=}\text{(}\text{}\text{, }\text{}\text{, }\text{}\text{, }\text{}\text{, }\text{}\text{, }\text{}\text{, }\text{}\text{, }\text{}\text{, }\text{}\text{, }\text{}\text{)}.$ The stable equilibrium point of the differential equations Eq.(S1) is $\overline{Z}=(\overline{z_{1}},\overline{z_{2}},\overline{z_{3}},\ldots,\overline{z_{10}})=(0,0,0,\ldots,0)$. The differential equations Eq.(S1) can be transformed into the difference equations $Z(k+1)=f(Z(k),p)$ using the Euler scheme [7] with a short time interval $\Delta t$. i.e.,

$\left\{ \begin{aligned} \boldsymbol{&}\boldsymbol{z}_{\boldsymbol{1}}\boldsymbol{(k+1)=}\boldsymbol{z}_{\boldsymbol{1}}\boldsymbol{(k)+}\left[ \frac{\boldsymbol{3}\left| \boldsymbol{P} \right|\boldsymbol{-}\boldsymbol{8}}{\boldsymbol{22}}\boldsymbol{+}\frac{\boldsymbol{8}\boldsymbol{-}\boldsymbol{3}\left| \boldsymbol{P} \right|}{\boldsymbol{22(1+}\boldsymbol{z}_{\boldsymbol{3}}\boldsymbol{(k))}}\boldsymbol{-}\frac{\boldsymbol{8+3}\left| \boldsymbol{P} \right|}{\boldsymbol{22}}\boldsymbol{z}_{\boldsymbol{1}}\boldsymbol{(k)+}\boldsymbol{\xi}_{\boldsymbol{1}}\boldsymbol{(k)} \right]\boldsymbol{\Delta k} \\ \boldsymbol{&}\boldsymbol{z}_{\boldsymbol{2}}\boldsymbol{(k+1)=}\boldsymbol{z}_{\boldsymbol{2}}\boldsymbol{(k)+}\left[ \frac{\boldsymbol{3}\left| \boldsymbol{P} \right|\boldsymbol{-}\boldsymbol{6}}{\boldsymbol{22}}\boldsymbol{+}\frac{\boldsymbol{6}\boldsymbol{-}\boldsymbol{3}\left| \boldsymbol{P} \right|}{\boldsymbol{22(1+}\boldsymbol{z}_{\boldsymbol{1}}\boldsymbol{(k))}}\boldsymbol{+}\frac{\boldsymbol{(6}\boldsymbol{-}\boldsymbol{3}\left| \boldsymbol{P} \right|\boldsymbol{)}\boldsymbol{z}_{\boldsymbol{3}}\boldsymbol{(k)}}{\boldsymbol{22(1+}\boldsymbol{z}_{\boldsymbol{3}}\boldsymbol{(k))}}\boldsymbol{-}\frac{\boldsymbol{6}}{\boldsymbol{11}}\boldsymbol{z}_{\boldsymbol{2}}\boldsymbol{(k)+}\boldsymbol{\xi}_{\boldsymbol{2}}\boldsymbol{(k)} \right]\boldsymbol{\Delta k} \\ \boldsymbol{&}\boldsymbol{z}_{\boldsymbol{3}}\boldsymbol{(k+1)=}\boldsymbol{z}_{\boldsymbol{3}}\boldsymbol{(k)+}\left[ \frac{\boldsymbol{3}\left| \boldsymbol{P} \right|\boldsymbol{-}\boldsymbol{8}}{\boldsymbol{22}}\boldsymbol{+}\frac{\boldsymbol{8}\boldsymbol{-}\boldsymbol{3}\left| \boldsymbol{P} \right|}{\boldsymbol{22(1+}\boldsymbol{z}_{\boldsymbol{1}}\boldsymbol{(k))}}\boldsymbol{-}\frac{\boldsymbol{8+3}\left| \boldsymbol{P} \right|}{\boldsymbol{22}}\boldsymbol{z}_{\boldsymbol{3}}\boldsymbol{(k)+}\boldsymbol{\xi}_{\boldsymbol{3}}\boldsymbol{(k)} \right]\boldsymbol{\Delta k} \\ \boldsymbol{&}\boldsymbol{z}_{\boldsymbol{4}}\boldsymbol{(k+1)=}\boldsymbol{z}_{\boldsymbol{4}}\boldsymbol{(k)+}\left[ \frac{\boldsymbol{3}\left| \boldsymbol{P} \right|\boldsymbol{-}\boldsymbol{10}}{\boldsymbol{22}}\boldsymbol{+}\frac{\boldsymbol{10}\boldsymbol{-}\boldsymbol{3}\left| \boldsymbol{P} \right|}{\boldsymbol{22(1+}\boldsymbol{z}_{\boldsymbol{1}}\boldsymbol{(k))}}\boldsymbol{+}\frac{\boldsymbol{(10}\boldsymbol{-}\boldsymbol{3}\left| \boldsymbol{P} \right|\boldsymbol{)}\boldsymbol{z}_{\boldsymbol{3}}\boldsymbol{(k)}}{\boldsymbol{22(1+}\boldsymbol{z}_{\boldsymbol{3}}\boldsymbol{(k))}}\boldsymbol{-}\frac{\boldsymbol{10}}{\boldsymbol{11}}\boldsymbol{z}_{\boldsymbol{4}}\boldsymbol{(k)+}\boldsymbol{\xi}_{\boldsymbol{4}}\boldsymbol{(k)} \right]\boldsymbol{\Delta k} \\ \boldsymbol{&}\boldsymbol{z}_{\boldsymbol{5}}\boldsymbol{(k+1)=}\boldsymbol{z}_{\boldsymbol{5}}\boldsymbol{(k)+}\left[ \frac{\boldsymbol{3}\left| \boldsymbol{P} \right|\boldsymbol{-}\boldsymbol{12}}{\boldsymbol{22}}\boldsymbol{+}\frac{\boldsymbol{(12}\boldsymbol{-}\boldsymbol{3}\left| \boldsymbol{P} \right|\boldsymbol{)}\boldsymbol{z}_{\boldsymbol{1}}\boldsymbol{(k)}}{\boldsymbol{22(1+}\boldsymbol{z}_{\boldsymbol{1}}\boldsymbol{(k))}}\boldsymbol{+}\frac{\boldsymbol{12}\boldsymbol{-}\boldsymbol{3}\left| \boldsymbol{P} \right|}{\boldsymbol{22(1+}\boldsymbol{z}_{\boldsymbol{3}}\boldsymbol{(k))}}\boldsymbol{-}\frac{\boldsymbol{12}}{\boldsymbol{11}}\boldsymbol{z}_{\boldsymbol{5}}\boldsymbol{(k)+}\boldsymbol{\xi}_{\boldsymbol{5}}\boldsymbol{(k)} \right]\boldsymbol{\Delta k} \\ \boldsymbol{&}\boldsymbol{z}_{\boldsymbol{6}}\boldsymbol{(k+1)=}\boldsymbol{z}_{\boldsymbol{6}}\boldsymbol{(k)+}\left[ \boldsymbol{-}\frac{\boldsymbol{4}}{\boldsymbol{11}}\boldsymbol{+}\frac{\boldsymbol{z}_{\boldsymbol{1}}\left( \boldsymbol{k} \right)}{\boldsymbol{11}\left( \boldsymbol{1+}\boldsymbol{z}_{\boldsymbol{1}}\left( \boldsymbol{k} \right) \right)}\boldsymbol{+}\frac{\boldsymbol{1}}{\boldsymbol{11}\left( \boldsymbol{1+}\boldsymbol{z}_{\boldsymbol{3}}\left( \boldsymbol{k} \right) \right)}\boldsymbol{+}\frac{\boldsymbol{2}}{\boldsymbol{11}\left( \boldsymbol{1+}\boldsymbol{z}_{\boldsymbol{5}}\left( \boldsymbol{k} \right) \right)}\boldsymbol{+}\frac{\boldsymbol{2}\boldsymbol{z}_{\boldsymbol{7}}\left( \boldsymbol{k} \right)}{\boldsymbol{11}\left( \boldsymbol{1+}\boldsymbol{z}_{\boldsymbol{7}}\left( \boldsymbol{k} \right) \right)}\boldsymbol{+}\frac{\boldsymbol{z}_{\boldsymbol{8}}\left( \boldsymbol{k} \right)}{\boldsymbol{11}\left( \boldsymbol{1+}\boldsymbol{z}_{\boldsymbol{8}}\left( \boldsymbol{k} \right) \right)}\boldsymbol{+}\frac{\boldsymbol{1}}{\boldsymbol{11(1+}\boldsymbol{z}_{\boldsymbol{9}}\boldsymbol{(k))}}\boldsymbol{-}\frac{\boldsymbol{14}}{\boldsymbol{11}}\boldsymbol{z}_{\boldsymbol{6}}\boldsymbol{(k)+}\boldsymbol{\xi}_{\boldsymbol{6}}\boldsymbol{(k)} \right]\boldsymbol{\Delta k} \\ \boldsymbol{&}\boldsymbol{z}_{\boldsymbol{7}}\boldsymbol{(k+1)=}\boldsymbol{z}_{\boldsymbol{7}}\boldsymbol{(k)+}\left[ \boldsymbol{-}\frac{\boldsymbol{1}}{\boldsymbol{11}}\boldsymbol{+}\frac{\boldsymbol{z}_{\boldsymbol{8}}\boldsymbol{(k)}}{\boldsymbol{11(1+}\boldsymbol{z}_{\boldsymbol{8}}\boldsymbol{(k))}}\boldsymbol{+}\frac{\boldsymbol{1}}{\boldsymbol{11(1+}\boldsymbol{z}_{\boldsymbol{9}}\boldsymbol{(k))}}\boldsymbol{-}\frac{\boldsymbol{16}}{\boldsymbol{11}}\boldsymbol{z}_{\boldsymbol{7}}\boldsymbol{(k)+}\boldsymbol{\xi}_{\boldsymbol{7}}\boldsymbol{(k)} \right]\boldsymbol{\Delta k} \\ \boldsymbol{&}\boldsymbol{z}_{\boldsymbol{8}}\boldsymbol{(k+1)=}\boldsymbol{z}_{\boldsymbol{8}}\boldsymbol{(k)+}\left[ \boldsymbol{-}\frac{\boldsymbol{1}}{\boldsymbol{11}}\boldsymbol{+}\frac{\boldsymbol{1}}{\boldsymbol{11(1+}\boldsymbol{z}_{\boldsymbol{9}}\boldsymbol{(k))}}\boldsymbol{-}\frac{\boldsymbol{19}}{\boldsymbol{11}}\boldsymbol{z}_{\boldsymbol{8}}\boldsymbol{(k)+}\boldsymbol{\xi}_{\boldsymbol{8}}\boldsymbol{(k)} \right]\boldsymbol{\Delta k} \\ \boldsymbol{&}\boldsymbol{z}_{\boldsymbol{9}}\boldsymbol{(k+1)=}\boldsymbol{z}_{\boldsymbol{9}}\boldsymbol{(k)+}\left[ \boldsymbol{-}\frac{\boldsymbol{1}}{\boldsymbol{11}}\boldsymbol{+}\frac{\boldsymbol{1}}{\boldsymbol{11(1+}\boldsymbol{z}_{\boldsymbol{8}}\boldsymbol{(k))}}\boldsymbol{-}\frac{\boldsymbol{19}}{\boldsymbol{11}}\boldsymbol{z}_{\boldsymbol{9}}\boldsymbol{(k)+}\boldsymbol{\xi}_{\boldsymbol{9}}\boldsymbol{(k)} \right]\boldsymbol{\Delta k} \\ \boldsymbol{&}\boldsymbol{z}_{\boldsymbol{10}}\boldsymbol{(k+1)=}\boldsymbol{z}_{\boldsymbol{10}}\boldsymbol{(k)+}\left[ \boldsymbol{-}\frac{\boldsymbol{1}}{\boldsymbol{11}}\boldsymbol{+}\frac{\boldsymbol{1}}{\boldsymbol{11(1+}\boldsymbol{z}_{\boldsymbol{8}}\boldsymbol{(k))}}\boldsymbol{+}\frac{\boldsymbol{3}\boldsymbol{z}_{\boldsymbol{9}}\boldsymbol{(k)}}{\boldsymbol{11(1+}\boldsymbol{z}_{\boldsymbol{9}}\boldsymbol{(k))}}\boldsymbol{-}\boldsymbol{2}\boldsymbol{z}_{\boldsymbol{10}}\boldsymbol{(k)+}\boldsymbol{\xi}_{\boldsymbol{10}}\boldsymbol{(k)} \right]\boldsymbol{\Delta k} \end{aligned} \right.$ (S2)

It is clear that $Z(k)$ is the vector of $Z(t)$ at the time instant $k\Delta t$. The Jacobian matrix of Eq.(S2) can be defined as $\left. J=\frac{\partial f(Z(k);p)}{\partial Z} \right|_{Z=\overline{Z}}$, where

$J=e^{\Delta t\bullet A}$ (S3)

With

$$A=\left[ \begin{matrix} -\frac{8+3\left| P \right|}{22} & 0 & \frac{3\left| P \right|-8}{22} & 0 & 0 & 0 & 0 & 0 & 0 & 0 \\ \frac{3\left| P \right|-6}{22} & -\frac{6}{11} & \frac{6-3\left| P \right|}{22} & 0 & 0 & 0 & 0 & 0 & 0 & 0 \\ \frac{3\left| P \right|-8}{22} & 0 & -\frac{8+3\left| P \right|}{22} & 0 & 0 & 0 & 0 & 0 & 0 & 0 \\ \frac{3\left| P \right|-10}{22} & 0 & \frac{3\left| P \right|-10}{22} & -\frac{10}{11} & 0 & 0 & 0 & 0 & 0 & 0 \\ \frac{12-3\left| P \right|}{22} & 0 & \frac{3\left| P \right|-12}{22} & 0 & -\frac{12}{11} & 0 & 0 & 0 & 0 & 0 \\ \frac{1}{11} & 0 & -\frac{1}{11} & 0 & -\frac{2}{11} & -\frac{14}{11} & \frac{2}{11} & \frac{1}{11} & -\frac{1}{11} & 0 \\ 0 & 0 & 0 & 0 & 0 & 0 & -\frac{16}{11} & \frac{1}{11} & -\frac{1}{11} & 0 \\ 0 & 0 & 0 & 0 & 0 & 0 & 0 & -\frac{19}{11} & -\frac{1}{11} & 0 \\ 0 & 0 & 0 & 0 & 0 & 0 & 0 & -\frac{1}{11} & -\frac{19}{11} & 0 \\ 0 & 0 & 0 & 0 & 0 & 0 & 0 & -\frac{1}{11} & \frac{3}{11} & -2 \end{matrix} \right]$$

From Eq.(S3), by taking $\Delta t=1$, we can obtain ten distinct eigenvalues with the largest eigenvalue ${0.76}^{|p|}$→1 when $p$→0. Therefore, the equilibrium point $\overline{Z}$ is stable when $p\in(0,1]$ and $p=0$ is a bifurcation point, at which the system undergoes a critical transition. We aim to detect early-warning signals that indicate the critical transition as a control parameter $p$ approaches a bifurcation point 0. According to MIWE method, for each simulation trial, we use the 29 samples generated when the control parameter $p$ is far away from the bifurcation point $p$ = 0 as the reference samples. Then in each simulation trial, based on the single sample $\{z_{1},z_{2}, \ldots, z_{29}\}$ derived for each parameter value $p\in[-0.5, 0.25]$, calculating the MIWE score according to the MIWE method is shown in Figure 2B of the main text, which accurately represents the warning signal before the imminent critical transition at p= -0.0001.

# Changes in local MIWE values for each gene in four real datasets


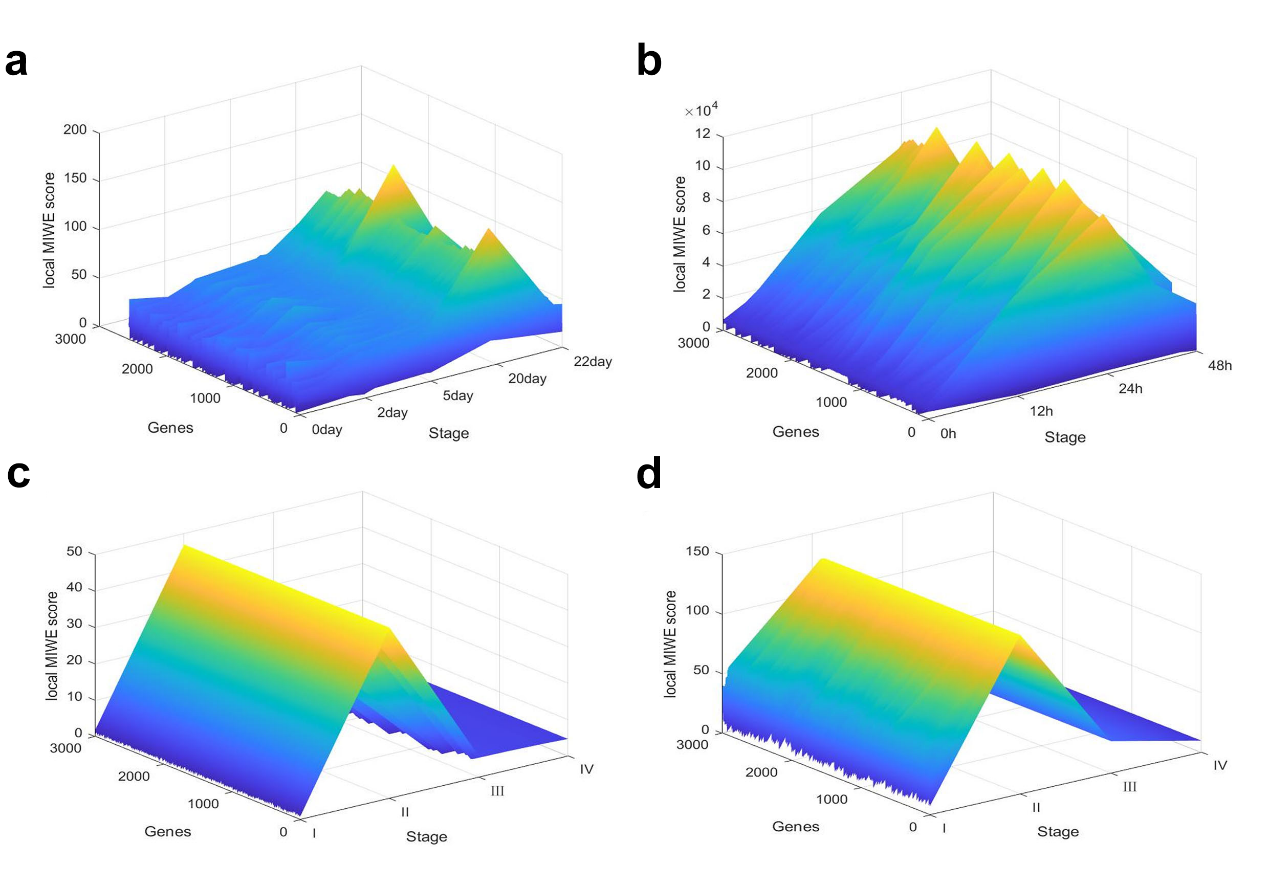


**Fig. S2.** **The landscape of local MIWE.** The values illustrates the dynamic evolution of network entropy in a global view for (a) MEF-to-neuron, (b) mESC to MP, (c) COAD, and (d) THCA, respectively.

# Functional annotation of TFs associated with embryonic differentiation from GSE79578 data

We select the top 20 TFs of regulatory signaling genes in GSE79578 data for analysis. The role of some TFs in cell differentiation and proliferation are shown in Table S1.

Table S1. Annotations of partial TFs functions of GSE79578

| Gene | Location | Family | Relation with embryonic differentiation progression |
| --- | --- | --- | --- |
| POU5F1 | cytosol | POU transcription factor | POU5F1 is a transcription factor and master regulator of cell pluripotency with indispensable roles in early embryo development and cell lineage specification [8]. |
| PRDM14 | nucleus | class V-like SAM-binding methyltransferase superfamily | PRDM14 plays important roles in primordial germ cell specification and embryonic stem cell pluripotency [9]. |
| BRF2 | nucleus | TFIIB | BRF2 activation contributes to cell growth and proliferation [10]. |
| KLF10 | nucleus | Sp1 C2H2-type zinc-finger protein | KLF10 inhibited cell proliferation, cell cycle progression and promoted apoptosis in vitro and in vivo [11]. |
| E2F6 | nucleus | E2F/DP | E2F6 is a member of the E2F family of TFs that control cell proliferation and cell fate [12]. |
| CTCF | nucleus | CTCF zinc-finger protein | CTCF is required for cell cycle regulation, embryonic development and formation of various adult cell types [13]. |

# Dynamic evolution of PPIs for two TCGA cancers: COAD, THCA


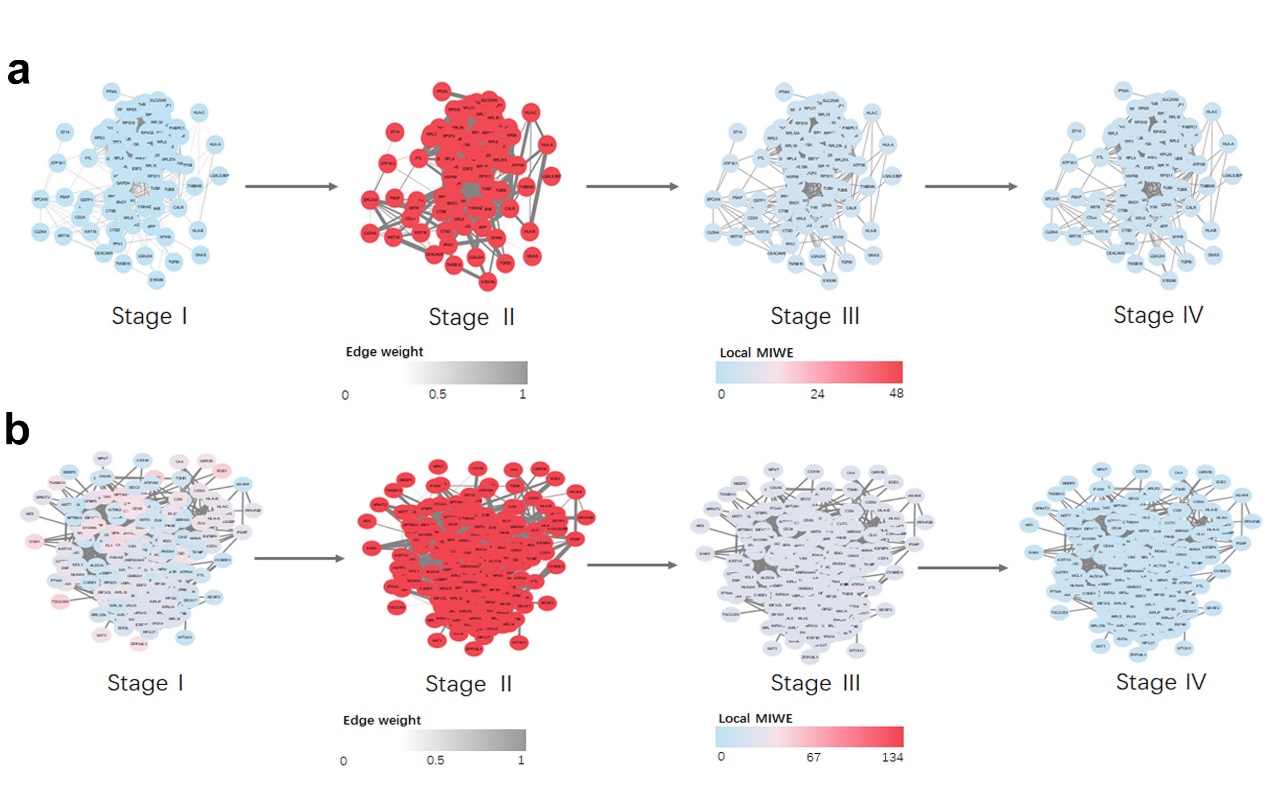


**Fig. S3. Dynamic PPI change.** The dynamic evolution of PPIs constructed by signaling genes of (a) COAD and (b) THCA.

#

# The Kaplan-Meier survival analysis of two cancer datasets

We use Kaplan-Meier survival analysis to verify the prediction results of the algorithm, and Log-Rank is used to test the significance of the survival curve. P value less than 0.05 indicated that the difference is statistically significant, and the survival time of patients diagnosed before the critical stage is longer than that of patients diagnosed after the critical stage.

In this study, survival analysis was applied to two kinds of cancer: COAD and THCA, shown in Fig. S4 as follows. There was no significant difference in the survival curve after the critical period (Fig. S4b,d), but the survival time of the samples before the transition period was much longer than that of the samples after the transition period. The earlier the disease was detected, the longer the survival time, and the higher the possibility of cure. These results show that our approach can effectively detect early warning signs during the progression of the disease that seriously affect the survival time of patients.


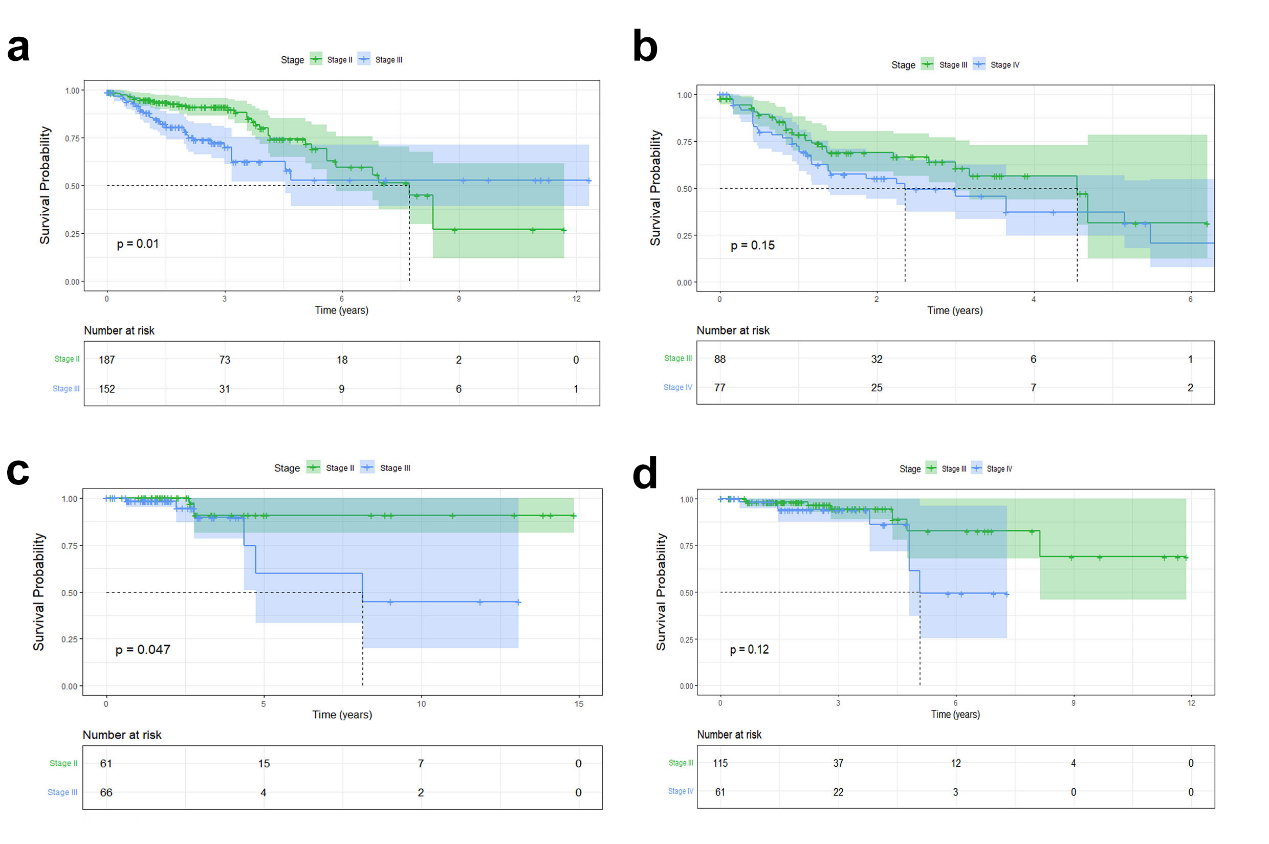


**Fig S4.** **Prognostic survival analysis.** (a) The survival curve of critical stage (II) of COAD vs that of the next stage of critical point (III) of COAD in survival analysis. (b) The survival curve of critical stage (III) of COAD vs that of the next stage of critical point (IV) of COAD in survival analysis. (c) The survival curve of critical stage (II) of THCA vs that of the next stage of critical point (III) of THCA in survival analysis. (d) The survival curve of critical stage (III) of THCA vs that of the next stage of critical point (IV) of THCA in survival analysis.

# Leave-one-out cross validation

We carry out leave-one-out cross validation by identifying DNBs for mESC to MP (GSE79578). The procedure is as follows:

(1) For mESC to MP, leaving a case sample $x_{i}$, $i$=1, 2, ……N out, we use the remaining N-1 case samples for identifying the DNBs based on the MIWE, and then calculated its MIWE score at each stage, i.e., ${MIWE}_{i}$, $i$=1, 2, ……N, where N represents the number of case samples.

(2) By using the identified DNBs, we carry out the validation for the excluded sample $x_{i}$ based on the MIWE. Finally, the average MIWE with the leave-one-out cross validation was obtained by $\sum_{i=1}^{N} {MIWE}_{i}/N$.

As shown in Fig S5, where red curves represent the average MIWE of the identified DNBs with all samples, and the green curves represent the average MIWE of the identified DNBs with leave-one-out cross validation. It can be seen that the average MIWE curves with leave-one-out cross validation have the same trends to those of the DNBs with all samples, which validates the effectiveness of the detected critical state.


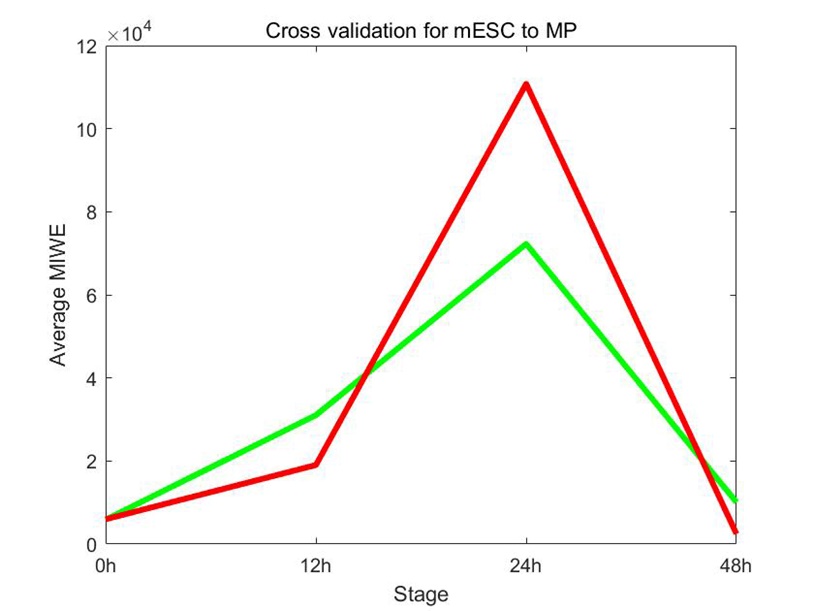


**Fig S5. Cross validation results for mESC to MP.** The red curves represent the average MIWE of the identified DNBs with all samples, and the green curves represent the average MIWE of the identified DNBs with leave-one-out cross validation.

# References

1. Cantone I, Marucci L, Iorio F, Ricci MA, Belcastro V, Bansal M, et al. A yeast synthetic network for in vivo assessment of reverse-engineering and modeling approaches. Cell. 2009;137:172–81.
2. Sherman MS, Cohen BA. Thermodynamic state ensemble models of cis-regulation. PLOS Computational Biology. 2012;8:e1002407.
3. Sueyoshi C, Naka T. Stability Analysis for the Cellular Signaling Systems Composed of Two Phosphorylation-Dephosphorylation Cyclic Reactions. Computational Molecular Bioscience. 2017;7:33-45.
4. Chen Y , Kim JK, Hirning AJ, Josić K, Bennett MR. Emergent genetic oscillations in a synthetic microbial consortium. Science. 2015;349:986-989.
5. Chen P, Li Y, Liu X, et al. Detecting the tipping points in a three-state model of complex diseases by temporal differential networks. J Transl Med. 2017;15:217.
6. Li C, Chen L, Aihara K. Stability of genetic networks with SUM regulatory logic: Lur’e system and LMI approach. IEEE Transactions on Circuits and Systems I: Regular Papers. 2006;53:2451-2458.
7. Woods CW, McClain MT, Chen MH, et al. A Host Transcriptional Signature for Presymptomatic Detection of Infection in Humans Exposed to Influenza H1N1 or H3N2. PLoS ONE. 2013;8:e52198.
8. Daigneault BW, Rajput S, Smith GW, et al. Embryonic POU5F1 is Required for Expanded Bovine Blastocyst Formation. Sci Rep. 2018;8:7753.
9. Lu Y, Wan Z, Zhang X, et al. PRDM14 inhibits 293T cell proliferation by influencing the G1/S phase transition. Gene. 2016;595:180-186.
10. Lockwood WW, Chari R, Coe BP, et al. Integrative genomic analyses identify BRF2 as a novel lineage-specific oncogene in lung squamous cell carcinoma. PLoS Med. 2010;7:e1000315.
11. Zhou M, Chen J, Zhang H, et al. KLF10 inhibits cell growth by regulating PTTG1 in multiple myeloma under the regulation of microRNA-106b-5p. Int J Biol Sci. 2020;16:2063-2071.
12. Dahlet T, Truss M, Frede U, et al. E2F6 initiates stable epigenetic silencing of germline genes during embryonic development. Nat Commun. 2021;12:3582.
13. Stik G, Vidal E, Barrero M, et al. CTCF is dispensable for immune cell transdifferentiation but facilitates an acute inflammatory response. Nat Genet. 2020;52:655-661.
